# What factors influence the choice of urban or rural location for future practice of Nepalese medical students? A cross-sectional descriptive study

**DOI:** 10.1186/s12960-015-0084-5

**Published:** 2015-11-10

**Authors:** Bhim Prasad Sapkota, Archana Amatya

**Affiliations:** Epidemiology and Disease Control Division, Department of Health Services, Ministry of Health and Population, Kathmandu, Nepal; Department of Community Medicine and Public Health Institute of Medicine, Tribhuvan University, Kathmandu, Nepal; Nepal Institute of Medical Sciences and Technology (NIMST), Kathmandu, Nepal

**Keywords:** Medical students in Nepal, Variables affecting their choice of urban versus rural practice settings as physician

## Abstract

**Background:**

Nepal is experiencing a public health issue similar to the rest of the world, i.e., the geographical maldistribution of physicians. Although there is some documentation about the reasons physicians elect to leave Nepal to work abroad, very little is known about the salient factors that influence the choice of an urban versus rural practice setting for those physicians who do not migrate. In recent years, around 1000 medical students became doctors within Nepal, but their distribution in rural locations is not adequate. The purpose of this study was to explore what factors influence the choice of urban or rural location for the future clinical practice of Nepalese medical students in the final year of their program

**Methods:**

A cross-sectional descriptive study design was used for this study involving Nepalese medical students in their final year of study and currently doing an internship in a medical college. The sample consisted of 393 medical students from four medical colleges in Nepal that were selected randomly. An anonymous self-administered questionnaire was used for data collection. To determine the association with rural location choice for their future practice setting, a comparison was done that involved demographic, socio-economic, and educational factors. Data were entered in EpiData and analyzed by using SPSS version 16.

**Results:**

Among the 393 respondents, two thirds were male (66.9%) and more than half were below 25 years of age. Almost all (93%) respondents were single and about two thirds (63.4%) were of Brahmin and Chhetri ethnic origin. About two thirds (64.1%) of the respondents were born in a rural setting, and 58.8% and 53.3% had a place of rearing and permanent address in a rural location, respectively. The predictors of future rural location choice for their clinical practice (based on the bivariate analysis) included:

- Rural (versus urban) place of birth, place of rearing, and permanent address
- Source of family income (service, business, and agriculture)
- Occupation of father (service, business and agriculture)
- Wealth ranking (higher, middle, and lower wealth rank)
- Educational factors: location, type of secondary education, and type of higher secondary education

**Conclusion:**

For medical students who were soon to complete their studies, demographic and educational factors were found to be significant predictors for a rural location choice, as opposed to socio-economic factors. Our findings indicate that to ensure the rural retention of physicians, the government of Nepal should attract potential medical students from those who were reared and educated in a rural setting.

## Background

One noted worldwide trend has been the migration of the health care worker, particularly physicians, from medically less served to better served areas. This paradoxical flow occurs over a continuum that includes both internal migration (usually from a rural to an urban setting) and external migration (from developing to developed countries), thereby creating a situation of limited availability and utilization of quality rural health care services [1]. This highly uneven distribution between urban and rural areas is rooted in the fact that cities offer workers better income, more opportunities for career progression, better infrastructure, and more social amenities than rural areas [2].

In addition to the uneven distribution of health care workers between urban and rural areas, a related issue is how to estimate the need for various types of health care personnel in a given geographical area. Countries should first identify their health problems in order to properly address their health worker needs, retention, recruitment, and training, if they are to come close to reaching the Millennium Development Goals (MDGs) for health [3].

A major challenge in the new millennium is the retention of health workers, not only in poorer countries but also within any country in remote and rural areas. Thus, an understanding of the factors that influence the decisions of health workers to leave (e.g., inadequate infrastructure, inadequate salaries, or intangibles) and the strategies effective for retention is imperative [4]. The phenomenon of inadequate numbers of doctors internationally, exacerbated by the large numbers of them in the private sector, in cities and in wealthier countries, has resulted in societal segments who are in dire need of help (e.g., poor, rural, and marginalized individuals and groups) and are underserved [5].

Turning now to the current situation regarding physician availability in Nepal, it is estimated that the doctor:population density in the capital city, Kathmandu, is 40 times that of rural Nepal [5]. Nepal has been working diligently to provide basic health services to people, particularly those living in remote/rural areas. However, in such locations, there are large numbers of vacant posts (47.3%) for medical officers in all districts [6]. Furthermore, even in places where these posts are filled on paper, their presence is questionable [7].

In summary, the health care system in Nepal is experiencing major challenges in regard to the availability of human resources, including physicians, for health care provision. In an excerpt from *The Kathmandu Post* (September 26, 2011), it was reported that that are just “two physicians per 10,000 population” (www.clubrunner.ca/Data/5360/951/…//FactsandStatsNepal.doc).

The factors behind the shortage and disparity in the distribution of doctors between urban to rural have not yet been studied in a systematic fashion [8]. Hence, there is a need to develop knowledge and understanding of the contributing factors that influence the decision of physicians to not go to, or remain in, rural clinical settings such as inadequate infrastructure, inadequate incentives (financial and nonfinancial), and related aspects [9]. The purpose of this study was to identify the predictors’ demographic-, economic-, and education-related factors for rural location choice for future practice among graduating medical students.

## Methods

### Research design

A cross-sectional, descriptive design was used for this study. To gain maximum knowledge about the research question, mixed methods were used, involving both a survey and a small qualitative component involving in-depth structured interviews

### Research setting and sampling strategy

The sample included (a) Nepalese medical students in their final year of study who were doing an internship in Bachelor of Medicine and Bachelor of Surgery (MBBS) at four (out of a total of 10) randomly selected colleges affiliated with Tribhuvan University and Kathmandu University and (b) recent medical student graduates/young doctors who were working in different geographical locations and/or preparing to leave for work abroad (*N* = 25). Sample size was calculated by using the following formula: *z*^2^*p q*/*L*^2^. The sample size was calculated to be 480. (For *p* = 50, *q* = 50, *L* = 5% and response rate of 80%) = 384 × 1.25 (for 80% response rate) = 480.

### Data collection and analysis

A hand-delivered, paper-based, self-administered anonymous questionnaire was used for data collection. Of the estimated 480 possible respondents, only 408 returned the questionnaire. Responses from 393 respondents were included because 15 questionnaires were incomplete and, thus, excluded from the study. Only those respondents who stated they planned to remain in Nepal to practice medicine following graduation (*N* = 260) were further asked to indicate whether they planned to work in a rural or an urban location they prefer to practice in after graduation.

The qualitative component involved three in-depth, structured interviews by the first author to obtain data from three recently graduated doctors working in a remote (hard-to-reach) and rural location. These data were subsequently entered into EpiData version 3.1 and transferred to SPSS version 16 for further analysis.

The predictor variables, such as demographic factors (except for ethnicity) and educational factors, were dichotomized and analyzed by cross-tabulation in 2 × 2 table.

Ethnicity and economic variables (i.e., source of family income, occupation of father, occupation of mother, and wealth ranks) were categorized into three categories, and odds ratio was calculated by using binary logistic regression.

Findings of the study were presented in frequency tables for univariate descriptive analysis. Bivariate findings were presented by cross-tabulation. Test of significance for association was assessed by using odds ratio with 95% confidence interval and *P* value of <0.05. Variables which were found to be significant for association with rural choice of future practice location in bivariate analysis were further analyzed through logistic regression only after testing of model fit. For test of model fit, Hosmer and Lemeshow chi-square test was applied. Written informed consent was taken from the respondents prior to the data collection. Confidentiality of the information was maintained. Ethical approval was taken from the ethical review board of the Institute of Medicine. Respondents’ autonomy was fully respected.

## Results

Of the 393 respondents, two thirds were male (66.9%), almost all (93.3%) were single, and more than half of them were below age 25 (mean: 24.49 years; standard deviation: 1.59 years). Their ethnic origins were as follows: about two thirds (63.4%) were Brahmin and Chhetri, one fourth (26.7%) were Janajati and Dalits, and a small percentage (9.9%) were from other cultural minority groups About two thirds (64.1%) of the participants were of rural birth place, 58.3% had been reared in a rural setting, and 53.3% had a rural setting as their permanent address.

Turning now to the socio-economic data findings, the majority of the respondents had their major source of family income in private and public services (54.7%), followed by the business sector (28.8%) and agriculture sector (16.5%). The occupational status of the mothers were as follows: about half (48.8%) were in the agricultural sector, followed by those in the service sector (32.1%). As for their fathers’ occupation, more than half (57%) were engaged in the service sector, followed by those in the business sector (29.3%).

A few points about the educational status of the parents of the respondents: whereas half (47.7%) of the mothers had an educational level below that of the secondary level, 32% had an educational level beyond that of a secondary level. About two thirds (64.4%) of the respondents’ fathers had above secondary level education. In the three wealth ranks, near equimodal distribution was found with the highest percentage (34.1%) in the middle wealth rank followed by the higher wealth rank (33.1%) and the lower wealth rank (32.8%).

About two thirds (66.2%) of the respondents had completed their secondary level education from private school whereas more than two thirds (70.5%) had completed their education from private college. Nearly two thirds (64.6%) of the respondents had completed their secondary education from an urban location while more than two thirds (71.5%) of the respondents had completed higher secondary education from an urban location. Less than one third (28%) of the respondents had scored distinction in secondary level education, whereas nearly the same respondents (29.8%) had scored distinction in higher secondary level education. Less than two thirds (60.8%) of the respondents have completed higher secondary education from higher secondary school as 10 + 2.

Among the 393 respondents, more than one fourth (27.7%) of the respondents perceived that higher education opportunities after 2 years work experience is the most influencing factor for choosing future practice location followed by training and skill improvement opportunities (22.4%),urban facilities (11.5%),supportive working environment (10.4%), and co-habitation with family members (10.2%).

### Choice of location

About two thirds (65.9%) of the respondents had chosen within-country location for future practice. Among those who had chosen a within-country choice, about an equal percentage of the respondents had chosen rural (50.8%) and urban (49.2%) location for their future practice after graduation (refer to Table 1).

**Table 1:** **Choice of practice location**

**Choice of location**	**Numbers**	**Percentage**
Within-country and out of country choice (*n* = 393)		
Within country	260	65.9
Out of country	133	34.1
Urban and rural choice (*n* = 260)		
Urban location	128	49.2
Rural location	132	50.8

### Association of predicting variables with rural location choice

Compared with the respondents who had been born in an urban location, those born in a rural location were four (odds ratio (OR) = 4.520) times more likely to chose a rural location for their future practice (*P* value <0.001 with 95% confidence interval (CI) = 2.526–8.086). In reference to the urban, respondents who had grown up in a rural location were found to be about six (OR = 5.720) times more likely to choose a rural location for their future practice (*P* value <0.001 with 95% CI = 3.239–10.10). Comparing with urban, respondents who were from a rural-located permanent address were found to be seven (OR = 6.979) times more likely to choose a rural location for their future practice (*P* value <0.001 with 95% CI = 3.979–12.240) (refer to Table 2).

**Table 2:** **Association of demographic characteristics with location choice (** ***n*** **= 260)**

**Demographic characteristics**	**Urban location** ***n*** **(%)**	**Rural location** ***n*** **(%)**	***P*** **value**	**Odds ratio**	**CI (95%)**
Sex of students					
Female	40 (47.1)	45 (52.9)	-	-	-
Male	88 (50.3)	87 (49.7)	0.625	0.879	0.523–1.476
Age of students					
Mean	24.35	24.64	0.123	NA	−0.064–0.079
S.D.	1.450	1.588			
Marital status					
Single	120 (50.2)	119 (49.8)	-	-	-
Married	8 (38.1)	13 (61.9)	0.287	1.639	0.655–4.097
Ethnic groups^a^					
Brahmin/Chhetri	82 (51.6)	77 (48.4)	-	-	-
Janajati	32 (43.2)	42 (56.8)	0.979	1.011	0.447–2.288
Dalits	14 (51.9)	13 (48.1)	0.443	1.413	0.584–3.421
Place of birth					
Urban	59 (73.8)	21 (26.2)	-	-	-
Rural	69 (38.3)	111 (61.7)	<0.001	4.520	2.526–8.086
Place of rearing^b^					
Urban	70 (75.3)	23 (24.7)	-	-	-
Rural	58 (34.7)	109 (65.3)	<0.001	5.720	3.239–10.10
Permanent address (at present)					
Urban	72 (68.6)	33 (31.4)	-	-	-
Rural	56 (36.1)	99 (63.9)	<0.001	6.979	3.979–12.240

^a^Nepalese caste system, CBS 2011

^b^Rearing up to the age of 10 years

Compared with agriculture, respondents whose family income was based on business were found to be less than one (OR = 0.322) times less likely to choose a rural location for future practice (*P* value 0.003 with 95% CI = 1.14–4.456). Compared with the above secondary level, respondents whose father had an educational attainment level below the secondary level were found to be less than one (OR = 0.452) times more likely to choose a rural location for future practice (*P* value 0.017 with 95% CI = 0.236–0.865). In reference to agriculture, respondents whose father’s occupation was business were found to have a twice (OR = 2.454) higher probability of selecting a rural location for their future practice (*P* value 0.016 with 95% CI = 1.185–5.070). In reference to above secondary level, respondents whose mothers had an educational attainment level of secondary level were two (OR = 2.348) times more likely to choose a rural location for future practice (*P* value 0.010 with 95% CI = 1.222–4.513). Compared with agriculture and housewife, respondents whose mother’s education was business were found to have a three (OR = 3.020) times higher probability of selecting a rural location for their future practice (*P* value <0.001 with 95% CI = 1.149–5.531). Compared to a lower wealth rank, respondents who were from middle wealth rank families had a two (OR = 2.045) times higher chance of selecting a rural location for future practice (*P* value 0.023 with 95% CI = 1.104–3.789) (refer to Table 3).

**Table 3:** **Association of Economic characteristics with location choice (n = 260)**

**Economic characteristics**	**Urban location** ***n*** **(%)**	**Rural location** ***n*** **(%)**	***P*** **value**	**Odds ratio**	**CI (95%)**
Sources of family income					
Agriculture	16 (31.4)	35 (68.6)	-	-	-
Business	44 (58.7)	31 (41.3)	0.003	0.322	1.14–4.456
Service and others	68 (50.7)	66 (49.3)	0.19	0.444	0.410–1.285
Education of father					
Above secondary (more than 10 years schooling)	93 (59.6)	63 (40.0)	-	-	-
Below secondary (less than 10 years schooling)	15 (27.8)	39 (72.2)	0.017	0.452	0.236–0.865
Secondary (10 years schooling)	20 (40)	30 (60)	0.189	1.733	0.762–3.941
Occupation of father					
Agriculture	13 (29.5)	31 (70.5)	-	-	-
Business	44 (57.9)	32 (42.1)	0.016	2.454	1.185–5.070
Service (private and public)	71 (50.7)	69 (49.3)	0.313	0.748	0.426–1.314
Education of mother					
Above secondary (more than 10 years schooling)	49 (62)	30 (38)	-	-	-
Below secondary (less than 10 years schooling)	48 (37.5)	80 (62.5)	0.684	0.863	0.424–1.756
Secondary (10 years schooling)	31 (58.5)	22 (41.5)	0.010	2.348	1.222–4.513
Occupation of mother					
Agriculture/housewife	22 (31)	49 (69)	-	-	-
Business	26 (52.2)	24 (48)	0.001	3.020	1.149–5.531
Service (private and public)	80 (57.6)	59 (42.4)	0.498	1.252	0.654–2.395
Wealth ranking					
Lower wealth rank	44 (42.3)	60 (57.7)	-	-	-
Middle wealth rank	42 (48.8)	44 (51.2)	0.023	2.045	1.104–3.789
Higher wealth rank	42 (60)	28 (40)	0.165	1.571	0.830–2.975

Compared to 10 + 2, respondents who had completed their higher secondary level education from I. Sc. were found to have a near to three (OR = 2.707) times higher chance of selecting a rural location for future practice (*P* value <0.001 with 95% CI = 1.632–4.490). In reference to the respondents who had completed their higher secondary education from private college, those who completed public college were three (OR = 3.068) times more likely to choose a rural location for their future practice (*P* value <0.001 with 95% CI = 1.786–5.271). Comparing with the respondents who had completed their higher secondary level education from an urban location, those from a rural location are three (OR = 3.611) times more likely to choose a rural location for future practice (*P* value <0.001 with 95% CI = 2.061–6.326) (refer to Table 4).

**Table 4:** **Association of educational characteristics with location choice (** ***n*** **= 260)**

**Educational characteristics**	**Urban location** ***n*** **(%)**	**Rural location** ***n*** **(%)**	***P*** **value**	**Odds ratio**	**CI (95%)**
School for secondary education					
Private school	97 (63.4)	56 (36.6)	-	-	-
Public school	31 (29)	76 (71)	<0.001	4.247	2.495–7.227
Location of secondary school					
Urban	103 (67.8)	49 (32.2)	-	-	-
Rural	25 (23.1)	83 (76.9)	<0.001	6.979	3.979–12.24
Performance in secondary level					
Distinction	39 (58.2)	28 (41.8)	-	-	-
Non-distinction	89 (46.1)	104 (53.9)	0.088	1.628	0.928–2.855
Higher secondary education					
10 + 2	87 (60)	58 (40)	-	-	-
I. Sc.	41 (35.7)	74 (64.3)	<0.001	2.707	1.632–4.490
Type of higher secondary college					
Private college	100 (58.5)	71 (41.5)	-	-	-
Public college	28 (31.5)	61 (68.5)	<0.001	3.068	1.786–5.271
Location of high. sec. college					
Urban	104 (59.1)	72 (40.9)	-	-	-
Rural	24 (28.6)	60 (71.4)	<0.001	3.611	2.061–6.326
Performance at higher sec. education					
Distinction	30 (60)	20 (40)	-	-	-
Non-distinction	98 (46.7)	112 (53.3)	0.090	1.714	0.915–3.210

One of the interesting findings from the qualitative study was that “Urban facilities, side job opportunities and preparation of higher study compel me to work in urban location- one of the recent graduates working in Kathmandu.” From this expression, it is obvious that expectation about side job opportunities is indicative towards the economic income while preparation for higher study is driven by professional advancement. Hence, confidence on economic income and professional advancement both are found to be associated with the choice of practice location among recent medical graduates.

Another significant finding from the qualitative study was that “lack of diagnostic facilities, lack of team work, political interference in decision making, high public expectation, frequent transfer and difficult life styles in the rural remote location are the hurdles to us for not to stay in rural location.” This statement reflects the need of creating a supportive working environment in rural remote districts to attract and retain the young doctors.

### Multivariate analysis

All variables which were found to be statistically significant for association with a rural location choice were further analyzed by logistic regression. For multivariate analysis, Hosmer and Lemeshow chi-square test for goodness of fit was applied for testing the model fit. It was found to be significant with a *P* value of 0.139 on putting the abovementioned 11 variables in the model.

Eleven variables were found to be significant in the bivariate analysis, i.e., place of birth, place of rearing, place of permanent address, source of family income, occupation of father, wealth ranking, type of secondary education, location of secondary education, type of higher secondary education, type of college for higher secondary level, and location of higher secondary education, but on the logistic regression, only two variables (i.e., place of rearing and location of secondary education) were found to be statistically significant for adjusted association by multivariate analysis (refer to Table 5).

**Table 5:** **Adjusted association of different factors with rural location choice**

**Variables**	**Unadjusted OR**	**AOR (95% CI)**	***P*** **value**
Place of birth			
Urban	-	-	-
Rural	4.520	0.755 (0.220–2.584)	0.655
Place of rearing			
Urban	-	-	-
Rural	5.720	4.520 (1.247–5.451)	0.021*
Permanent address			
Urban	-	-	-
Rural	6.979	0.740 (0.285–1.921)	0.537
Source of family income			
Agriculture	-	-	-
Business	0.322	1.326 (0.281–6.265)	0.722
Service (public and private)	0.444	1.248 (0.270–5.771)	0.777
Occupation of father			
Agriculture	-	-	-
Business	2.454	1.327 (0.250–7.041)	0.740
Service (public and private)	0.748	0.689 (0.154–3.082)	0.626
Wealth ranking			
Lower wealth rank	-	-	-
Middle wealth rank	2.045	1.651 (0.807–3.379)	0.170
Higher wealth rank	1.571	1.451 (0.655–3.214)	0.359
Type of school for secondary education			
Private school	-	-	-
Public school	4.247	1.704 (0.851–3.409)	0.132
Location of secondary education			
Urban location	-	-	-
Rural location	6.979	3.706 (1.787–7.687)	<0.001*
Type of higher secondary education			
10 + 2	-	-	-
I. Sc.	2.707	1.415 (0.723–2.770)	0.311
College for higher sec. education			
Private college	-	-	-
Public college	3.068	1.149 (0.532–2.481)	0.724
Location of high. secondary education			
Urban location	-	-	-
Rural location	3.611	0.540 (0.203–1.435)	0.217

The symbol *Means it is statistically significant.

### Place of rearing

Compared to urban place of rearing, respondents who had been reared in a rural location were found to be four (adjusted OR = 4.520) times more likely to be associated with choice of rural location for future practice (*P* value 0.021 with 95% CI = 1.247–5.451).

### Location of secondary education

Compared with the urban location of secondary education, respondents who had completed their secondary education from a rural location are nearly four (adjusted OR = 3.706) times more likely to be associated with a choice of rural location (*P* value <0.001 with 95% CI = 1.787–7.687).

The regression equation for rural location choice in the form of *Y* = *C* + *b*_1_*x*_1_ + *b*_2_*x*_2_ +_…_ was found to be as follows:

$$ \begin{array}{l}\mathrm{Rural}\kern0.5em \mathrm{location}\kern0.5em \mathrm{choice}=\left(-1.394\right)+1.628\left(\mathrm{place}\kern0.5em \mathrm{of}\mathrm{rearing}\right)+\\ {}1.682\left(\mathrm{location}\kern0.5em \mathrm{of}\kern0.5em \mathrm{secondary}\kern0.5em \mathrm{education}\right)\end{array} $$

## Discussion

The study was conducted with the objective of identifying the predictors for choice of future practice location among graduating medical students in Nepal. Demographic, economic, education, and job-related variables were assessed as predictors.

Age distribution of the respondents was found to be a mean age of 24.49 years with standard deviation of 1.517 years. About two thirds (66.9%) of the respondents were male. This finding reflects the gender-wise disproportionate access to medical education. Moreover, it is also consistent with the findings of Nepal living standards survey 2010/11 which showed that the general literacy rate among males is 71.6% while as that of females is 44.5% [10]. Nearly two thirds (63.4%) of the respondents were from the Brahmin/Chhetri ethnic group; this may be due to the higher literacy rate among the Brahmin and Chhetri ethnic group compared to the Janajati and Dalits. About two thirds (64%) of the respondents were found to be from a rural place of birth which may be due to the higher proportion (83%) of the general population residing in rural locations as per the preliminary findings of census 2011 [11]. More than half (54%) of the respondents’ family income was based on service followed by business (28.8%) and agriculture (16.5%). About two thirds (64.4%) of the respondents’ fathers had above secondary level of education. From this finding, we can assume that there may be a positive association of father’s education with major source of family income.

Around two thirds (65.9%) of the respondents had chosen the within-country location for future practice as it was similar with the findings from the study of Nick Simons Institute and the Institute of Medicine in which 63.1% of the graduates were found to be working within Nepal during the period of 2008 to 2010 [1].

Among 10 job-related attributes for future practice location, more than one in four (27.7%) respondents had given first priority to higher education opportunity; similarly, more than one in five (22.4%) respondents had given first priority to training and skill improvement opportunities. Combining these two factors, we can conclude that more than 50% of the respondents gave first priority for better professional outcome while choosing future practice location. Similar findings were found in the study entitled “Future practice preference among medical students in Ghana in 2010” [12]. The findings of the study stated that medical students prefer to work in a rural location if there is better opportunity for higher education in the subject of their interest and skill improvement opportunities through regular training.

One of the recently graduated doctors stated in an in-depth interview that “my father have been working as teacher in a rural village where there is poor access to minimum health services. He had a dream to make me a doctor and to serve the same district. To make his dream true I have decided to work there in government health facility at the district.” A rural-located place of rearing was near to two times more likely to be associated with choosing a rural location. This finding is partially consistent with the findings from “A study on professional expectation of medical students in Angola, Guinea Bissau and Mozambique in 2011” which showed that 44.4% of the medical students were interested to work in the public sector of which around two thirds (63.4%) were from a rural-located permanent address [13].

Contradictory findings of the study was that around an equal number of the respondents had chosen the urban (49.2%) and rural (50.8%) location for future practice, but in the findings of the Nick Simons Institute, it was found that only one among three graduates were found as working in Kathmandu valley.

In the current study, rural place of birth, rural place of rearing, and rural permanent address were found to be statistically significant for association with a rural location choice. The findings were consistent with the findings of a USA-based study in 1974 entitled “Choice of location for practice of medical school graduates” which revealed that rural place of birth, rural place of rearing, and rural location of permanent address of the respondents were three times more likely to choose a rural location [14]. But the findings were contradictory with the study results of the “Factors influencing family physicians to enter rural practice: Does rural or urban background makes the difference?” a study in Canada in 2005. The Canadian study results revealed that “two thirds of the rural physicians weren’t from rural background” [15]. But consistent findings were found in the study entitled “Do south African rural origin medical students return to rural practice?” which revealed that more than 40% of the rural-origin graduates were in rural practice compared to 5% of the urban-origin graduates [16].

Among various economic factors, business as a source of family income and parent’s occupation, as well as middle wealth rank of the family, were found to be associated with rural location choice. But the business as source of family income showed the inverse relation with rural location choice. It may be due to the factor that the students from the family having business as a source of income might give more priority to monetary income and that working in rural settings may not fulfill their will. Regarding the findings, no more results were found on these factors.

Among education-related variables, rural location for secondary and higher secondary education was associated with rural location choice. This may be due to their familiarity with the rural environment. Public school and college for secondary and higher secondary level education were also associated with rural location choice. It may be confounded due to the economic status of the family. Level of performance in higher secondary level education was also found to be associated with a rural location which may be due to the higher level of confidence among those who scored distinction in higher secondary education. The background variables related to demographic factors and educational factors were more significant than economic factors. To ensure rural retention of doctors, the government should attract the students from a rural place of rearing and rural secondary education for medical education. At the same time of publishing the report of this research in 2011, a newly established medical college by the Nepal government (Patan Academy of Health Sciences) has started to enroll students having a rural rearing and rural schooling in MBBS.

## Conclusion

For rural location choice, rural location of secondary education and rural location of rearing were found to be associated as ultimate predictors on adjusted association. Other factors like place of birth, permanent address, source of family income, occupation of father, wealth ranking, type of secondary education, and type of higher secondary education were found as confounders.

Since the study was a cross-sectional and descriptive type, the association observed in the study cannot be assumed as being a causal relation. To establish the causal relationship between these variables and the choice of future practice as within country and rural location, further analytical studies should be conducted.

## Abbreviations

AOR: adjusted odds ratio
CI: confidence interval
HRH: human resources for health
I. Sc.: Intermediate of Science
MBBS: Bachelor of Medicine and Bachelor of Surgery
NA: not applicable
OR: odds ratio
